# Change of weight status during school age and its association with late adolescent blood pressure: Results from a 15-year longitudinal study in China

**DOI:** 10.3389/fpubh.2022.980973

**Published:** 2022-08-19

**Authors:** Xijie Wang, Yanhui Dong, Sizhe Huang, Bin Dong, Jun Ma, Wannian Liang

**Affiliations:** ^1^Vanke School of Public Health, Tsinghua University, Beijing, China; ^2^Institute for Healthy China, Tsinghua University, Beijing, China; ^3^Institute of Child and Adolescent Health & School of Public Health, Peking University, Beijing, China; ^4^Zhongshan Health Care Center for Primary and Secondary Schools, Zhongshan, China

**Keywords:** longitudinal analysis, school-aged children, high blood pressure (HBP), epidemiology, overweight and obese

## Abstract

**Background:**

Change in obesity risk could be related to shift in high blood pressure (HBP) risk, while individualized influence of weight change on high blood pressure is in need of exploration.

**Methods:**

A total of 16,446 children (53.47% boys) and 13,9021 effective annual measurements from 2006 to 2020 were recruited. Children's weight status, both at baseline and endpoint, was categorized as underweight, normal, overweight, and obese according to the age and sex-specific Body Mass Index z scores. HBP at late adolescence was defined with the last two measurements for each child. Populational attributable risk (PAR) of weight trait on HBP risk was calculated.

**Results:**

Compared to children who maintained normal weight during follow-up, staying obese was associated with the highest HBP risk with OR of 6.39 (95% CI: 4.46, 9.15; *p* < 0.001) and PAR of 28.71% (95% CI: 21.58, 35.54) in boys, and OR of 6.12 (95% CI: 2.80, 13.37; *p* < 0.001) and PAR of 12.75% (95% CI: 4.29, 21.02) in girls. Returning from obese to normal weight was associated with lowered HBP risk, with ORs of 1.07 (95% CI: 0.69, 1.66; *p* = 0.771) in boys and 0.73 (95% CI: 0.25, 2.12; *p* = 0.566) in girls.

**Conclusion:**

Weight loss program could be effective to reduce HBP risk during school age, while the underlying mechanism needs further exploration.

## Introduction

China has become one of the highest-ranking countries in both percentage increase and predicted numbers of overweight children. Results from the recent nationwide survey conducted in 2014 showed that 14% of school-aged children were overweight, while another 6.4% of the children were obese (1). By 2025, 48.5 million of overweight children and millions of obesity-linked comorbidities are expected (2). Even mild obesity was found to be associated with the loss of one in ten potential disease-free years during middle and later adulthood, (3) and the effect of excess weight on years of life lost with the greatest magnitude were young individuals (4). To relieve the massive burden on both economy and healthcare systems caused by overweight and obesity (5), early screening and management of high-risk populations was always of great importance, which aligned with the interests of public health professionals.

In the pediatric population, hypertension is by far the major cardiovascular risk factor associated with overweight and obesity (6). From a worldwide perspective, the prevalence of childhood hypertension has increased for decades, making it much more common and a growing public health challenge in the general pediatric population. The pooled estimated prevalence of hypertension was 4.0% in 2019 among individuals 19 years and younger worldwide, (7) while the domestic prevalence among school-aged children in China has risen from 4.4% in 2005 to 6.4% in 2014 (8). Being the leading preventable risk factor for cardiovascular disease and all-cause mortality worldwide (9, 10), exhaustive prevention strategies of hypertension and overweight were still in need of exploration.

Apart from well-established evidence which indicates the strong association of childhood obesity occurrence and late-life hypertension, change in weight status from longitudinal observation study, with the outstanding advantages in capturing the dynamic growth change during school age, has become a new research spot. Pooled cohort study found that greater obesity duration was associated with worse blood pressure outcomes in mid-adulthood (11). Retrospective cohort studies from China and the United States found that both children who maintained a high level of body mass index (BMI) and those who transit from non-obese to obese status during childhood and adolescence would experience elevated hypertension risk during follow-up (12–14). Population-based studies also found that the peak of hypertension and weight status transition both occurred around the age of growth spurt, (13, 15) while pooled analysis found that the prevalence of hypertension peaked at 7.89% among those aged 14 years (7). However, most of the previous studies were conducted on the basis of population study; individualized analysis of weight change on high blood pressure (HBP) risk was still limited.

In the present study, a longitudinal follow-up data from 2006 to 2020 were constructed to explore: (1) how did the change in weight status relate to the blood pressure profile at late adolescence (aged 16–18), and (2) whether returning to normal weight status would offset the HBP risk at late adolescence.

## Methods

### Study setting

Data of the present study were extracted from the 2006 to 2020 annual physical examinations of primary, middle, and high school students in Zhongshan, China. Generally, all school-aged children from public schools were asked to attend a physical examination once every year, from grade 1 of primary school to grade 3 of high school. Ideally, each child would have 12 examinations with the time interval between adjacent examinations of ~1 year. All measurements were conducted by qualified medical physicians under the management of Zhongshan Health Care Center for Primary and Secondary School, while the equipment and working team of physical examination were relatively fixed.

### Study population

Among all the children who attended school physical examinations from 2006 to 2020, a total of 16,446 children who met the following criteria were included for the present analysis: (1) had at least 8 examinations; (2) had the first examination before 9 years of age; (3) had at least one measurement at late adolescence, which was the age between 16 and 18 years; (4) with full records of sex and date of birth. The baseline of this retrospective cohort was when participants had their very first physical examination, while the endpoint was when they had their last physical examination, which occurred at 16–18 years of age. Use of this data in the present study has been authorized by Zhongshan Health Care Center for Primary and Secondary School. All the information linked with individual privacy had been removed from the dataset before authorization, therefore the data of the present study was completely anonymous. The present analysis has been approved by the institution review board of Peking University (No. IRB00001052-20011-exempt).

### Data collection

Demographic characteristics, including sex, date of birth, and residence classification (urban or rural) were collected at enrollment for physical examinations in primary school, and were recorded to the registration system. Date information was generated for each of the examinations, while the age (in year) at each examination was calculated as (date of examination-date of birth)/365.25.

Height was measured with portable stadiometers (model TZG, China) and recorded to the nearest 0.1 centimeters. Weight was measured with level-type weight scale (model RGT-140, China) and recorded to the nearest 0.1 kilogram. Children would wear light undergarments and stand barefoot for these measurements. BMI was calculated as weight (kg)/height^2^(m^2^).

Blood pressure, including systolic blood pressure (SBP) and diastolic blood pressure (DBP), were measured with mercury sphygmomanometer (model XJ11D, China), stethoscopes (model TZ-1, China) and appropriate cuffs. Blood pressure was measured at least twice from the right arm, while children were asked to sit quietly for at least 5 min before measurement. Extra measurements were carried if the difference of either SBP or DBP exceeded 10 mmHg. The average of both SBP and DBP were calculated with the last two measurements.

All the above measurements were conducted in strict compliance with the Administrative Measures for Physical Examinations for Primary and Secondary School Students, which was published by National Ministry of Education and National Health Commission.

### Main exposure and outcome assessment

Main exposure of the present study was the weight status of each child at both baseline and endpoint. Weight status, categorized as underweight, normal, overweight and obesity, were evaluated based on the age- and sex-specific BMI z score according to the growth reference from World Health Organization (16). Children who were normal weight at both baseline and endpoint were set as the reference group for all analysis.

Blood pressure at endpoint, both continuously and categorical, were the main outcome of the present study. HBP was defined as age-, sex- and height-specific systolic blood pressure (SBP) or diastolic blood pressure (DBP) ≥ 95^th^ percentile for baseline measurements, and blood pressure ≥ 130/80 mmHg for endpoint measurements, according to the 2017 version of Clinical Practice Guideline for Screening and Management of High Blood Pressure in Children and Adolescents (17). Since the blood pressure data were derived from single-time measurement, which may be insufficient to determine HBP, BP measurements of the first two examinations were used to define baseline HBP and those of the last two examinations were used to define endpoint HBP, in order to avoid overestimation of HBP prevalence. For example, one participant would be determined as HBP at baseline only if their BP levels at their first two examinations were all categorized as HBP.

### Statistical analysis

Continuous descriptive statistics were displayed as mean and standard deviations, while categorical variables were displayed as numbers and percentages. Linear regression models were conducted to analyze the difference of SBP and DBP, while log-binomial regression models were conducted to analyze the difference in HBP risk among children of varying weight status at baseline and endpoint. Population attributable risk of weight status change on HBP risk was calculated based on the log-binomial regression model.

As significant sex disparities existed in prevalence of both overweight and HBP, all analysis were conducted separately by sex. Baseline HBP status, age of overweight/obesity onset, and residence were involved for adjusted models.

All analysis were performed with Stata version 14.0 (StataCorp LP, College Station, TX). Associations were considered as significant when two-tailed *P* < 0.05.

## Results

A total of 16,446 children (53.5% boys), along with 139,021 measurements from 2006 to 2020, were included in the present analysis. There were 9,309 (56.6%) of the children that lived in urban areas. The mean age was 7.12 (SD: 0.49) at baseline and 17.02 (SD: 0.59) at endpoint, with a mean follow-up time of 9.90 (SD: 0.65) years. The combined prevalence of overweight and obesity was 12.4% at baseline and 11.5% at endpoint. The prevalence of HBP at endpoint was 18.0% in boys and 5.2% in girls, respectively. The descriptive statistics of participants were displayed in Table 1, while the sample distribution of each year was in Supplementary Table e1.

**Table 1:** Descriptive statistics of participants at baseline and at endpoint.

**Characteristics**	**Total**	**Boys**	**Girls**	***P-*value**
**Baseline**				
Number, *n* (%)	16,446	8,794 (53.5)	7,652 (46.5)	-
Residence, urban, *n* (%)	9,309 (56.6)	4976 (56.6)	4,333 (56.6)	0.957
Age, years, mean (SD)	7.12 (0.49)	7.13 (0.48)	7.11 (0.49)	0.006
SBP, mmHg, mean (SD)	109.99 (8.57)	110.72 (8.64)	109.15 (8.41)	<0.001
DBP, mmHg, mean (SD)	68.36 (6.47)	68.64 (6.63)	68.04 (6.27)	<0.001
HBP, *n* (%)	3,332 (20.3)	2,087 (23.7)	1,245 (16.3)	<0.001
**Weight status**				<0.001
Normal, *n* (%)	13,544 (82.4)	6,854 (78.0)	6,690 (87.4)	
Overweight, *n* (%)	1,345 (8.2)	847 (9.6)	498 (6.5)	
Obesity, *n* (%)	698 (4.2)	518 (5.9)	180 (2.4)	
Underweight, *n* (%)	859 (5.2)	575 (6.5)	284 (3.7)	
**Endpoint**				
Age, years, mean (SD)	17.02 (0.59)	17.04 (0.60)	17.00 (0.59)	0.003
Follow-up time, years, mean (SD)	9.90 (0.65)	9.90 (0.65)	9.89 (0.64)	0.104
**Weight status**				<0.001
Normal, *n* (%)	13,754 (83.6)	7,001 (79.6)	6,753 (88.3)	
Overweight, *n* (%)	1,442 (8.8)	1,012 (11.5)	430 (5.6)	
Obesity, *n* (%)	444 (2.7)	342 (3.9)	102 (1.3)	
Underweight, *n* (%)	806 (4.9)	439 (5.0)	367 (4.8)	
SBP, mmHg, mean (SD)	117.72 (11.75)	122.74 (10.43)	111.97 (10.47)	<0.001
DBP, mmHg, mean (SD)	68.38 (7.17)	69.02 (7.57)	67.64 (6.75)	<0.001
HBP, *n* (%)	1,976 (12.0)	1,580 (18.0)	396 (5.2)	<0.001

*SBP, systolic blood pressure; DBP, diastolic blood pressure. High blood pressure was defined based on the 2017 version clinical practice guideline for screening and management of high blood pressure in children and adolescents*.

Children who remained obese during follow-up generally had the highest BP level, as well as HBP prevalence, at endpoint. A total of 99 boys and 13 girls who remained obese were defined as HBP, with the correspondence prevalence of 56.3 and 30.2%, respectively. Children who remained a normal weight or underweight generally had lower BP level at endpoint. For those who stayed normal weight, SBP was 121.93 (SD: 10.06) mmHg for boys and 111.48 (SD: 10.19) mmHg for girls, while DBP was 68.67 (SD: 7.26) mmHg for boys and 67.48 (SD: 6.65) mmHg for girls, respectively. The correspondent HBP prevalence was 15.4% in boys and 4.2% in girls (Table 2).

**Table 2:** Blood pressure at late adolescence of children with different weight status.

**Baseline status**	**Endpoint status**	**Boys**			**Girls**		
		**SBP**	**DBP**	**HBP**	**SBP**	**DBP**	**HBP**
Normal	Normal	121.93 ± 10.06	68.67 ± 7.26	916 (15.4)	111.48 ± 10.19	67.48 ± 6.65	257 (4.2)
	Overweight	126.77 ± 9.27	69.91 ± 7.79	136 (24.6)	117.03 ± 9.45	68.67 ± 7.13	20 (8.6)
	Obesity	130.94 ± 7.33	72.17 ± 8.17	25 (35.2)	124.19 ± 11.37	70.90 ± 8.11	4 (19.1)
	Underweight	120.31 ± 10.63	68.65 ± 7.53	25 (9.3)	111.50 ± 10.48	68.29 ± 6.81	15 (5.2)
Overweight	Normal	123.50 ± 9.90	68.40 ± 7.21	99 (20.7)	113.18 ± 10.87	67.64 ± 6.76	25 (7.7)
	Overweight	128.38 ± 9.48	71.36 ± 8.15	95 (34.7)	119.52 ± 9.75	69.26 ± 6.93	20 (14.6)
	Obesity	130.87 ± 8.63	72.26 ± 8.19	42 (44.2)	123.97 ± 8.55	69.76 ± 7.59	9 (23.7)
	Underweight	NA	NA	NA	NA	NA	NA
Obesity	Normal	125.22 ± 9.56	69.46 ± 7.63	33 (21.0)	111.37 ± 9.91	66.71 ± 7.12	4 (5.1)
	Overweight	129.13 ± 9.38	70.26 ± 8.32	73 (39.5)	120.53 ± 12.53	70.15 ± 8.41	18 (30.5)
	Obesity	133.63 ± 8.96	72.57 ± 8.33	99 (56.3)	124.47 ± 8.75	73.00 ± 9.04	13 (30.2)
	Underweight	NA	NA	NA	NA	NA	NA
Underweight	Normal	116.69 ± 10.51	69.18 ± 7.45	29 (7.2)	107.45 ± 10.35	67.02 ± 6.44	6 (2.9)
	Overweight	NA	NA	NA	NA	NA	NA
	Obesity	NA	NA	NA	NA	NA	NA
	Underweight	116.42 ± 10.72	68.29 ± 7.47	8 (4.5)	108.87 ± 11.06	67.70 ± 6.82	5 (6.5)

*SBP and DBP were displayed as mean ± standard error; HBP was displayed as number (%).*

*SBP, systolic blood pressure; DBP, diastolic blood pressure; HBP, high blood pressure; NA, not applicable*.

Compared to children who remained a normal weight, children who remained overweight or obese had the highest increase of SBP and DBP level, with a difference of 10.41 (95% CI: 8.94, 11.88) mmHg for obese boys and 11.36 (95% CI: 8.33, 14.39) mmHg for obese girls in SBP. Children who transitioned from lower to higher weight level generally had increased BP level, while those who remained normal or underweight had the lowest BP level (Table 3).

**Table 3:** Difference in blood pressure at late adolescence among children of different weight status.

**Baseline status**	**Endpoint status**	**Boys**		**Girls**	
		**SBP**	**DBP**	**SBP**	**DBP**
Normal	Normal	0 (reference)	0 (reference)	0 (reference)	0 (reference)
	Overweight	3.35 (2.42, 4.27)^***^	1.16 (0.46, 1.86)^**^	4.26 (2.84, 5.68)^***^	1.10 (0.17, 2.04)^*^
	Obesity	7.52 (5.20, 9.84)^***^	3.43 (1.69, 5.18)^***^	10.81 (6.47, 15.16)^***^	2.94 (0.07, 5.82)^*^
	Underweight	−1.22 (−2.42, −0.02)^*^	0.08 (−0.82, 0.99)	0.09 (−1.09, 1.28)	0.81 (0.03, 1.60)^*^
Overweight	Normal	0.89 (−0.03, 1.81)	−0.37 (−1.07, 0.32)	1.04 (−0.11, 2.19)	0.07 (−0.69, 0.83)
	Overweight	5.35 (4.16, 6.55)^***^	2.39 (1.49, 3.29)^***^	6.95 (5.23, 8.67)^***^	1.41 (0.28, 2.55)^*^
	Obesity	8.19 (6.21, 10.18)^***^	3.38 (1.88, 4.87)^***^	11.22 (8.00, 14.44)^***^	1.82 (−0.31, 3.94)
	Underweight	NA	NA	NA	NA
Obesity	Normal	2.40 (0.84, 3.95)^**^	0.57 (−0.60, 1.75)	−1.01 (−3.26, 1.25)	−1.10 (−2.59, 0.39)
	Overweight	6.08 (4.64, 7.52)^***^	1.29 (0.20, 2.37)^*^	7.03 (4.43, 9.63)^***^	1.83 (0.11, 3.55)^*^
	Obesity	10.41 (8.94, 11.88)^***^	3.47 (2.35, 4.58)^***^	11.36 (8.33, 14.39)^***^	4.86 (2.86, 6.86)^***^
	Underweight	NA	NA	NA	NA
Underweight	Normal	−4.72 (−5.71, −3.73)^***^	0.59 (−0.15, 1.34)	−3.89 (−5.29, −2.49)^***^	−0.46 (−1.38, 0.46)
	Overweight	NA	NA	NA	NA
	Obesity	NA	NA	NA	NA
	Underweight	−4.89 (−6.38, −3.40)^***^	−0.22 (−1.34, 0.90)	−2.88 (−5.14, −0.61)^*^	0.13 (−1.36, 1.63)

*Adjusted for baseline high blood pressure, age of overweight/obesity onset and residence (urban/rural).*

*SBP, systolic blood pressure; DBP, diastolic blood pressure; HBP, high blood pressure; NA, not applicable.*

* *P <0.05;*

** *P <0.01;*

*** *P <0.001*.

For children who were underweight either at baseline or at endpoint, the relative risk of HBP at endpoint was lower than children who remained normal weight, with ORs ranged between 0.33 (95% CI: 0.16, 0.70; *p* = 0.004) and 1.41 (95% CI: 0.53, 3.77; *p* = 0.491). In boys, staying obese led to the highest HBP risk, with the OR of 6.39 (95% CI: 4.46, 9.15; *p* < 0.001) and correspondent PAR of 28.71% (95% CI: 21.58, 35.54). Change from normal or overweight to obese also led to significant increase in HBP risk, with ORs of 2.67 (95% CI: 1.50, 4.76; *p* = 0.001) and 4.56 (95% CI: 2.82, 7.36; *p* < 0.001), and the correspondent PAR of 13.05% (95% CI: 3.91, 22.04) and 22.25% (95% CI: 13.33, 30.80), respectively. HBP risk in girls who changed from lower to higher weight categories showed similar trends as that in boys. Children who returned from obese or overweight to normal weight had much lower HBP risk compared to those who remained overweight or obese. In boys, the ORs for children moving from obesity and overweight to normal weight were 1.07 (95% CI: 0.69, 1.66; *p* = 0.771) and 1.24 (95% CI: 0.95, 1.62; *p* = 0.113), respectively, while that in girls were 0.73 (95% CI: 0.25, 2.12; *p* = 0.566) and 1.65 (95% CI: 1.03, 2.64; *p* = 0.037), respectively. Change of HBP risk in girls was similar to the pattern we found in boys (Table 4).

**Table 4:** Risk and populational attributable risk of high blood pressure at late adolescent from children of different weight status.

**Baseline status**	**Endpoint status**	**Boys**		**Girls**	
		**OR (95% CI)**	**PAR**	**OR (95% CI)**	**PAR**
Normal	Normal	1 (reference)	0 (reference)	1 (reference)	0 (reference)
	Overweight	1.30 (1.01, 1.69)^*^	3.07 (−0.06, 6.20)	1.67 (0.94, 2.97)	2.37 (−0.72, 5.46)
	Obesity	2.67 (1.50, 4.76)^**^	13.08 (3.91, 22.04)	3.07 (0.87, 10.88)	6.38 (−3.42, 16.05)
	Underweight	0.64 (0.41, 1.00)	−4.42 (−8.41, −0.41)	1.29 (0.73, 2.28)	1.07 (−1.52, 3.65)
Overweight	Normal	1.24 (0.95, 1.62)	2.46 (−0.70, 5.61)	1.65 (1.03, 2.64)^**^	2.30 (−0.19, 4.79)
	Overweight	2.28 (1.68, 3.09)^***^	10.67 (6.16, 15.13)	2.62 (1.51, 4.55)^**^	5.19 (1.32, 9.04)
	Obesity	4.56 (2.82, 7.36)^***^	22.25 (13.33, 30.80)	4.86 (1.99, 11.86)^**^	10.38 (1.70, 18.90)
	Underweight	NA	NA	NA	NA
Obesity	Normal	1.07 (0.69, 1.66)	0.73 (−4.24, 5.69)	0.73 (0.25, 2.12)	−1.06 (−4.28, 2.16)
	Overweight	2.97 (2.08, 4.25)^***^	14.83 (9.01, 20.55)	4.87 (2.52, 9.43)^***^	10.40 (4.00, 16.71)
	Obesity	6.39 (4.46, 9.15)^***^	28.71 (21.58, 35.54)	6.12 (2.80, 13.37)^***^	12.75 (4.29, 21.02)
	Underweight	NA	NA	NA	NA
Underweight	Normal	0.46 (0.31, 0.70)^***^	−6.99 (−10.06, −3.91)	0.68 (0.29, 1.60)	−1.27 (−3.73, 1.19)
	Overweight	NA	NA	NA	NA
	Obesity	NA	NA	NA	NA
	Underweight	0.33 (0.16, 0.70)^**^	−9.18 (−13.63, −4.68)	1.41 (0.53, 3.77)	1.50 (−3.29, 6.28)

*Adjusted for baseline high blood pressure, age of overweight/obesity onset and residence (urban/rural).*

*OR, odds ratio; CI, confidential interval; PAR, populational attributable risk; NA, not applicable.*

* *P <0.05;*

** *P <0.01;*

*** *P <0.001*.

## Discussion

In this longitudinal follow-up study with 16,446 school-aged children, we were able to describe weight trait during this critical period of growth and development. Results from the present analysis confirmed that children who were overweight and obese during school-age would have the highest risk of HBP at late adolescence. And we further found that returning to normal weight could help to reduce the HBP risk to even as low as that of children who stayed normal weight during school age.

Overweight and obesity, with an estimated domestic prevalence of ~25% in children (18, 19) and over 30% in adults, (20) has become a major threat to the general population health. Controlling strategies of school-aged overweight and obesity has been held and improved for decades in China, with the latest prevention and control implementation plan being published in 2020 (21). However, the health effects related to weight control was seldomly reported from long-term observational studies, especially from Asian countries.

Previous studies of our team, on the basis of population-based analysis, found that children who started overweight during pubertal height growth had higher risk of HBP at late adolescence, compared to those who remained overweight during school-age (13). In the present analysis with individual-based exploration, we confirmed the previous findings, and further found that returning from obese to overweight, as well as from overweight to normal weight, would both lead to decreased HBP risk at late adolescence. Returning to normal weight could even lead to substantially similar HBP risk to children who maintained a normal weight status. Weight control is known to play important roles in therapeutic approaches of hypertension (22–24). The Avon Longitudinal Study of Parents and Children had reported normal pulse wave velocity in obese children reverting to normal fat mass indices at the age of 17, (25) however the change of blood pressure was not reported. While long-term observational studies are insufficient in children and adolescents to determine how much of the disease burden could be reduced by weight control strategies, more research is required to understand which specific intervention components are most effective and in whom, for what health outcome, and how best to maintain intervention effects (26). Despite the effect of weight loss on HBP control, health workers should always keep in mind that the best way to prevent any cardiovascular disease is to avoid the occurrence of excessive weight, rather than post-obesity intervention (27). Meanwhile, the reference guidelines used for HBP definition and evaluation also mattered in estimating its prevalence, and would therefore influence the effect size of weight change on HBP outcome (28) which should be kept in mind in exploration of the present findings.

The main strength of the present study was the longitudinal follow-up design over a large and stable school-aged population with regular measurement intervals. The setting of this population provided reliable measurement results and enabled us to explore the change in weight status and its association with blood pressure outcomes during school age.

However, there are a few limitations to be noted for the present analysis. First, participants in this study were from a city in southeastern China with low obesity prevalence. Any estimates in the present study may underestimate the relationship between obesity and HBP. Second, as the physiological changes were relatively complicated during this special period, timing of pubertal maturation, (29) as well as endocrine changes during pubertal growth, (30) may have critical roles in weight change. The underlying mechanism by which weight loss reduced the risk of HBP are desired in future study in order to develop cost-effective obesity intervention strategies. Third, as the relationship between obesity and hypertension was cliché but complicated, more health behavior factors, longer follow-up time including preschool age, (31) and more diverse indicators are needed for further exploration on the impact of overweight, obesity, and their mechanism toward elevated hypertension risk.

## Conclusion

Returning from overweight or obese to normal weight during school age could help to reduce the risk of HBP at late adolescence. However, its underlying mechanism needs further exploration.

## Data availability statement

The data analyzed in this study is subject to the following licenses/restrictions: The data would not be made public at the request of the data authorizer. Requests to access these datasets should be directed to JM, majunt@bjmu.edu.cn.

## Ethics statement

The studies involving human participants were reviewed and approved by the Institution Review Board of Peking University (No. IRB00001052-20011-exempt). Written informed consent from the participants and participants legal guardian/next of kin was not required to participate in this study in accordance with the national legislation and the institutional requirements.

## Author contributions

XW, BD, and JM contributed to conception and design of the study. YD, SH, and BD organized the database. XW performed the statistical analysis and wrote the first draft of the manuscript. BD, JM, and WL gave critical revision to the manuscript. All authors contributed to manuscript revision, read, and approved the submitted version.

## Funding

The present study was supported by the National Natural Science Foundation (81673194 to JM and 81903344 to BD). The funder did not participate in the work.

## Conflict of interest

The authors declare that the research was conducted in the absence of any commercial or financial relationships that could be construed as a potential conflict of interest.

## Publisher's note

All claims expressed in this article are solely those of the authors and do not necessarily represent those of their affiliated organizations, or those of the publisher, the editors and the reviewers. Any product that may be evaluated in this article, or claim that may be made by its manufacturer, is not guaranteed or endorsed by the publisher.
