# Supplementary material for: Change of weight status during school age and its association with late adolescent blood pressure: Results from a 15-year longitudinal study in China
Source: Front Public Health. 2022 Aug 19;10:980973. doi: 10.3389/fpubh.2022.980973 (PMC9437432; doi:10.3389/fpubh.2022.980973)
Supplement: Supplementary file 1 [file Table_1.DOCX]

| eTable 1. Distribution of study participants by year and sex | | | |
| --- | --- | --- | --- |
| Research year | Person-time | | |
|  | Total | Boys | Girls |
| 2006 | 265 | 133 | 132 |
| 2007 | 8,495 | 4,513 | 3,982 |
| 2008 | 7,892 | 4,247 | 3,645 |
| 2009 | 11,960 | 6,423 | 5,537 |
| 2010 | 14,461 | 7,692 | 6,769 |
| 2011 | 14,246 | 7,551 | 6,695 |
| 2012 | 14,155 | 7,500 | 6,655 |
| 2013 | 14,589 | 7,786 | 6,803 |
| 2014 | 7,079 | 3,839 | 3,240 |
| 2015 | 10,158 | 5,269 | 4,889 |
| 2016 | 15,048 | 7,950 | 7,098 |
| 2017 | 13,801 | 7,394 | 6,407 |
| 2018 | 3,131 | 1,784 | 1,347 |
| 2019 | 2,780 | 1,480 | 1,300 |
| 2020 | 961 | 501 | 460 |
| Total | 139,021 | 74,062 | 64,959 |
